# Electrolyte Imbalance in Acute Traumatic Brain Injury: Insights from the First 24 h

**DOI:** 10.3390/clinpract14050141

**Published:** 2024-08-30

**Authors:** Alina Săcărescu, Mihaela-Dana Turliuc

**Affiliations:** 1 Department of Medical Specialties III, “Grigore T. Popa” University of Medicine and Pharmacy, Universității 16, 700115 Iași, Romania; 2 Department of Neurology, Clinical Rehabilitation Hospital, Pantelimon Halipa 14, 700661 Iași, Romania; 3 Department of Surgery II, “Grigore T. Popa” University of Medicine and Pharmacy, Universitătii 16, 700115 Iași, Romania; mihaela.turliuc@umfiasi.ro; 4 Department of Neurosurgery I, “Prof. Dr. N. Oblu” Clinical Emergency Hospital, Ateneului 2, 700309 Iași, Romania

**Keywords:** traumatic brain injury, electrolytes, early monitoring, Glasgow Coma Scale, chloride, potassium

## Abstract

Background/Objectives: Electrolyte disturbances are common in patients with traumatic brain injury (TBI), particularly affecting sodium, potassium, chloride, and calcium levels. This study aims to provide insights into these disturbances within the first 24 h post-injury. Methods: We conducted a cross-sectional analysis of 50 TBI patients, excluding those with conditions affecting electrolyte balance. Electrolyte levels were measured, and correlations with demographic data, trauma mechanisms, imaging findings, and Glasgow Coma Scale (GCS) scores were analyzed. Results: The results indicated that chloride levels inversely correlated with GCS scores (ρ = −0.515; *p* = 0.002), suggesting that elevated chloride may indicate severe neurological impairment. Potassium levels were significantly associated with subdural hematoma (*p* = 0.032) and subarachnoid hemorrhage (*p* = 0.043), highlighting their potential as markers for severe brain injuries. No significant associations were found between sodium or calcium levels and the studied variables. Conclusions: These findings underscore the importance of early monitoring of chloride and potassium levels in TBI patients to improve management and outcomes. Future research should focus on larger, multi-center studies to validate these findings and develop comprehensive guidelines for managing electrolyte imbalances in TBI patients.

## 1. Introduction

Patients with traumatic brain injury (TBI) commonly experience electrolyte disturbances, which can present as abnormalities in sodium, potassium, chloride, and calcium levels, among others [1,2,3]. Changes in serum sodium levels are the most common and critical electrolyte abnormality, with both hyponatremia and hypernatremia being possible manifestations [4]. Hypernatremia, affecting 16% to 40% of TBI patients, typically manifests within the initial days post-injury due to factors like diabetes insipidus, hyperosmolar therapy, dehydration, and iatrogenic causes, and it is associated with higher mortality and longer hospital stays [5,6,7]. Hyponatremia, affecting about 13.2% of TBI patients, often arises from the syndrome of inappropriate antidiuretic hormone secretion (SIADH), or cerebral salt-wasting syndrome (CSWS) within the first week, with higher risk linked to severe injury, low Glasgow Coma Scale (GCS) scores, cerebral edema, or basal skull fractures [8,9,10,11,12].

Hyperkalemia affects 17.77% of TBI patients and often arises during the initial resuscitative period, with a period prevalence of 29% within the first 12 h after admission in patients with non-crush trauma [4,13]. This condition may follow an initial phase of hypokalemia and primarily results from catecholamine release, blood product transfusions, pharmacological agents such as succinylcholine, acidosis, and tissue ischemia [14,15,16]. Hypokalemia usually appears immediately after injury and peaks within the first few days, primarily resulting from catecholamine-induced potassium shifts, renal potassium loss, fluid deficits, and hypothermia. Younger patients and those with severe injuries are especially vulnerable, facing additional complications and increased mortality [14,16,17,18,19].

Calcium and chloride disturbances are also significant in TBI. Hypercalcemia can result from prolonged immobility or worsen with underlying hyperparathyroidism [20,21]. Hypocalcemia, common early post-injury and exacerbated by transfusions, is linked to severe coagulopathy and higher mortality, requiring careful management [22,23,24]. Hyperchloremia can develop acutely within the initial days post-TBI, coinciding with the hyperemia phase (days 1–3) marked by increased cerebral blood flow and decreased arterio-jugular venous oxygen differences [25,26]. Hypochloremia has also been proved to occur in severe TBI cases and appears to be associated with increased mortality [27].

Electrolyte changes are crucial when treating patients with TBI. Although some studies have focused on these changes and the mechanisms through which they occur, there is a notable lack of data regarding changes within the first 24 h post-injury. The rationale for focusing on the 24 h window post-injury is based on the fact that early electrolyte disturbances may reflect the initial physiological impact of brain injury and offer valuable prognostic information. By examining electrolyte changes within this critical period, we aim to identify imbalances that could indicate injury severity and guide timely clinical interventions. Specifically, this study investigates whether acute-phase electrolyte disturbances correlate with clinical outcomes such as GCS scores, imaging findings, and the need for surgery. We hypothesize that early electrolyte abnormalities may be linked to more severe injuries, lower GCS scores, and a higher likelihood of surgical intervention. While supported by existing literature, the acute-phase focus of our study seeks to provide new insights into these associations. By concentrating on the immediate post-injury period, this study aims to fill a gap in the literature and enhance our understanding of early electrolyte disturbances as potential biomarkers for guiding TBI management decisions.

## 2. Materials and Methods

This cross-sectional study involved 50 patients admitted for TBI at the Emergency Hospital “Prof. Dr. Nicolae Oblu” in Iași within 24 h of experiencing the trauma. As a regional trauma center, patients were admitted at varying times after the injury, with some patients being found unconscious for unknown durations of time (less than a day) and others being transferred from smaller local hospitals where initial stabilization took place. To maintain data relevance, we included only those admitted within 24 h of the trauma. Patients with a history of diabetes mellitus, thyroid or adrenal dysfunctions, recent trauma (within the last six months), or disorders/injuries of the hypothalamus or pituitary gland were excluded. Exclusion criteria further encompassed underage or pregnant patients and those with severe anemia defined by hemoglobin levels under 8 g/dL.

All data on electrolyte levels (sodium, potassium, ionized calcium, chloride), measured upon admission to our hospital and within the first 24 h of the trauma, were collected from medical charts. All blood samples were processed identically in the laboratory of the Emergency Hospital “Prof. Dr. Nicolae Oblu” using standard procedures. Normal electrolyte levels, as defined by the hospital’s laboratory, are 136–145 mmol/L for sodium, 3.5–5.1 mmol/L for potassium, 1.15–1.33 mmol/L for ionized calcium, and 98–107 mmol/L for chloride. Additional data, including demographic details, trauma mechanism, GCS scores, imaging results, and surgical needs, were extracted from medical records. Trauma mechanisms were categorized as unknown, falls from the same level, falls from a height, traffic accidents, aggression, and others. All patients in this study underwent routine non-contrast computed tomography (CT) scans as part of their standard clinical management for TBI. We specifically assessed the CT scans for relevant findings, including basal skull fractures, other skull fractures, subdural hematomas, epidural hematomas, subarachnoid hemorrhages, cerebral hemorrhages, and, in cases of suspected diffuse axonal injury (DAI), hemorrhages at characteristic locations such as the gray–white matter junction, corpus callosum, and brainstem. Given the limited sensitivity of CT for detecting non-hemorrhagic lesions indicative of DAI, magnetic resonance imaging (MRI) was performed in cases where clinical suspicion of DAI remained high despite inconclusive CT findings. MRI provided greater sensitivity for detecting both hemorrhagic and non-hemorrhagic lesions in these characteristic areas. TBI severity was categorized by GCS scores: mild (GCS 13-15), moderate (GCS 9-12), and severe (GCS 3-8).

Statistical analyses were conducted using IBM SPSS Statistics for Windows, Version 26.0 (IBM Corp, Armonk, NY, USA), and G*Power software (Version 3.1.9.7) was used for power analysis calculation. To identify associations between variables Spearman correlation coefficients, the Kruskal–Wallis test, independent-sample T test, Mann–Whitney U test, Q-Q Plot, and Shapiro–Wilk test were utilized, with significance set at *p* < 0.05. The study received ethical approval from the local committees of the Emergency Hospital “Prof. Dr. Nicolae Oblu” Iași (4281/10.03.2020) and “Grigore T. Popa” University of Medicine and Pharmacy Iași (14/5.10.2020).

## 3. Results

**Figure 1:**
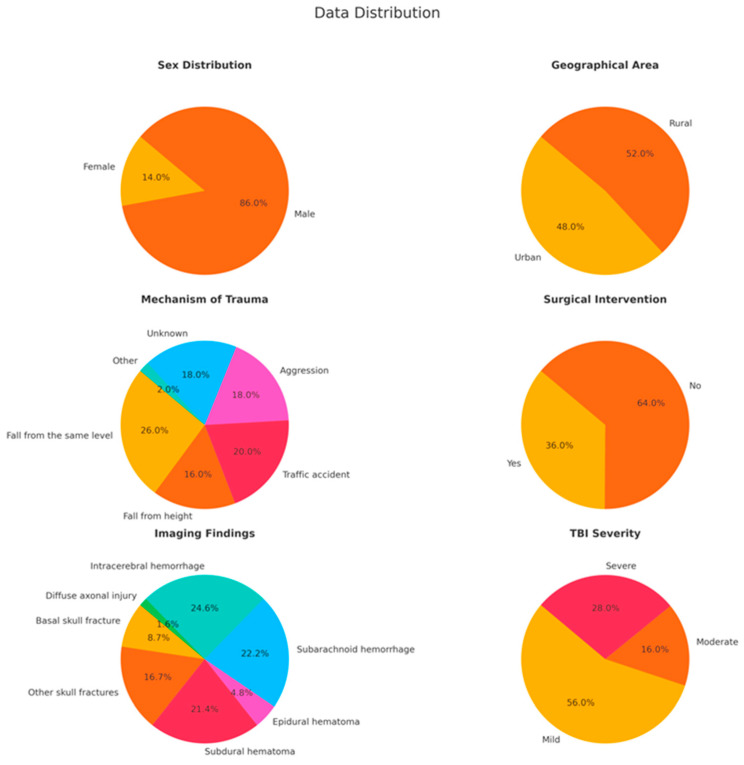
The key characteristics of the study population are represented in these pie charts, showing sex and geographical distributions, mechanisms of trauma, the need for surgical intervention, imaging findings, and severity of TBI. The data indicate a male majority, an almost equal urban–rural split, and a high occurrence of severe injuries such as subarachnoid hemorrhage and intracerebral hemorrhage.

Among the 50 patients included in the study, 43 (86%) were male and 7 (14%) were female, with a mean age of 51.48 years, ranging from 18 to 86 years. Figure 1 provides a summary of the key characteristics of the study population.

The mean levels of electrolytes were as follows: sodium was 138.5 mmol/L (median = 139.6 mmol/L, range = 113.6–145.7 mmol/L), potassium was 3.90 mmol/L (median = 3.88 mmol/L, range = 2.90–5.10 mmol/L), ionized calcium was 1.03 mmol/L (median = 1.04 mmol/L, range = 0.84–1.21 mmol/L), and chloride was 104.53 mmol/L (median = 105.4 mmol/L, range = 80.3–114.5 mmol/L). The Shapiro–Wilk test and Q-Q Plots confirmed a normal distribution for all these electrolytes in the overall group. Electrolyte disturbances were observed across varying severities of TBI and surgical interventions. Hyponatremia and hypocalcemia were the most common disturbances, affecting 16% and 64% of patients, respectively. Hypokalemia and hyperchloremia were also noted, affecting 12% and 22% of patients, respectively. No cases of hyperkalemia or hypercalcemia were observed. The full distribution of electrolyte imbalances, their prevalence, and their associations with TBI severity and surgical interventions are detailed in Table 1. 

To compare electrolyte concentrations between urban and rural areas, normality was reassessed for each geographic area. Sodium and chloride were not normally distributed, so the Mann–Whitney U test was used. The test revealed no significant difference in serum sodium levels between urban (mean rank = 22.48) and rural (mean rank = 22.52) patients (U = 241.5, *p* = 0.991). Similarly, no significant difference in serum chloride levels was found between urban (mean rank = 14.71) and rural (mean rank = 19.44) patients (U = 97.0, *p* = 0.159). For potassium and calcium, which were normally distributed, independent-sample t-tests were conducted. No significant difference was found for potassium levels between urban (mean = 3.92 mmol/L) and rural (mean = 3.90 mmol/L) patients (t (42) = 0.113, *p* = 0.910, 95% CI [−0.283, 0.317], df = 42). Similarly, there was no significant difference in calcium levels between urban (mean = 1.041 mmol/L) and rural (mean = 1.038 mmol/L) patients (t (36) = 0.108, *p* = 0.914, 95% CI [−0.058, 0.064], df = 36).

To compare electrolyte concentrations between males and females, normality was reassessed by sex. Sodium and chloride were not normally distributed, so the Mann–Whitney U test was applied. The test revealed no significant difference in serum sodium levels between males (mean rank = 22.86) and females (mean rank = 20.57) (U = 116.0, *p* = 0.664). Similarly, no significant difference in serum chloride levels was observed between males (mean rank = 16.36) and females (mean rank = 20.60) (U = 52.0, *p* = 0.365). For potassium and calcium, which were normally distributed, independent-sample t-tests were conducted. A significant difference in potassium levels was found between males (mean = 3.99 mmol/L) and females (mean = 3.51 mmol/L; t (42) = 2.500, *p* = 0.016, 95% CI [0.091, 0.856], df = 42). For calcium, no significant difference was observed between males (mean = 1.0387 mmol/L) and females (mean = 1.0430 mmol/L; t (36) = −0.103, *p* = 0.919, 95% CI [−0.088, 0.080], df = 36). 

**Figure 2:**
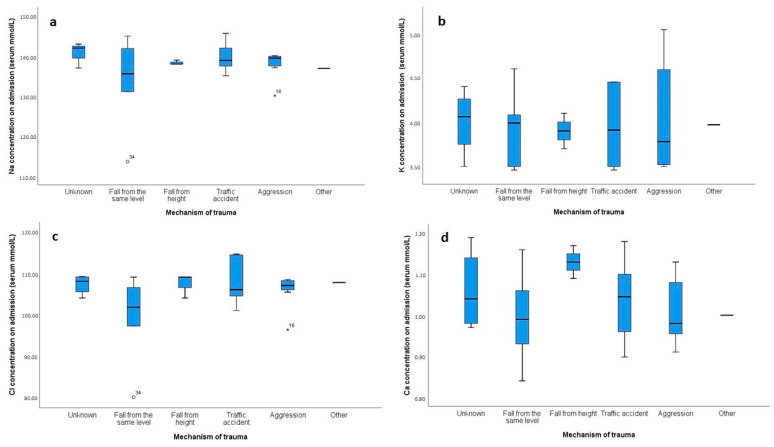
Box plots showing the distribution of electrolyte concentrations (mmol/L) on admission across different mechanisms of trauma. Panel (**a**) represents sodium (Na) concentrations, (**b**) shows potassium (K) concentrations, (**c**) depicts chloride (Cl) concentrations, and (**d**) illustrates calcium (Ca) concentrations. The categories for the mechanism of trauma include unknown, fall from the same level, fall from height, traffic accident, aggression, and other. Outliers are represented by circles, and extreme outliers are indicated by stars.

The distribution of electrolyte levels across different mechanisms of trauma was assessed using box plots (Figure 2). Notable variability and outliers were observed, particularly in the sodium (Figure 2a) and chloride (Figure 2c) concentrations for categories such as “aggression” and “traffic accident”. To formally assess the normality of these distributions, a Shapiro–Wilk test was conducted. The results indicated that sodium and chloride levels did not follow a normal distribution for several trauma mechanisms. Specifically, for sodium, the normality was rejected for “aggression” (*p* = 0.004) and “fall from height” (*p* < 0.001), and for chloride, normality was rejected for “aggression” (*p* = 0.002) and “fall from height” (*p* < 0.001). These findings, along with the observed variability in the box plots, suggested that non-parametric methods, such as the Kruskal–Wallis test, were more appropriate for comparing electrolyte levels across different trauma mechanisms due to the deviations from normality. The Kruskal–Wallis tests revealed no significant differences in the distribution of sodium (*p* = 0.427), potassium (*p* = 0.944), chloride (*p* = 0.251), or calcium (*p* = 0.936) concentrations across the different trauma mechanisms.

**Figure 3:**
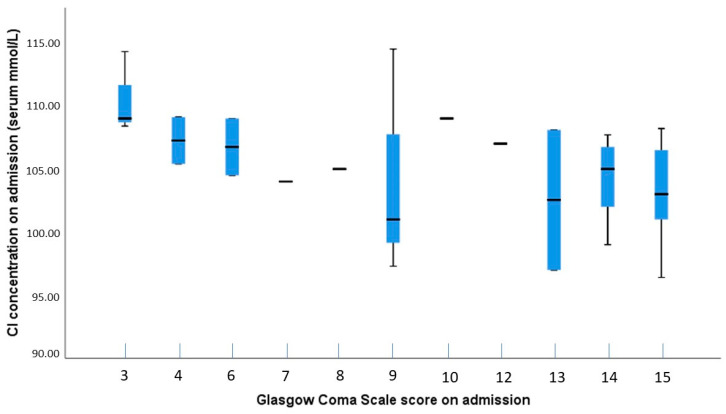
Boxplot showing the distribution of chloride concentrations by GCS scores on admission. The plot illustrates variability in chloride levels across different GCS scores, with greater fluctuations and outliers observed at lower GCS scores, suggesting a potential relationship between chloride concentration and the severity of TBI.

Possible correlations between electrolyte levels and GCS scores were analyzed using the Spearman test, which showed no significant results for sodium (*p* = 0.054), potassium (*p* = 0.985), and ionized calcium (*p* = 0.738). A significant negative correlation was found between chloride levels and GCS scores (ρ = −0.515; *p* = 0.002), suggesting a moderate to large effect size (Figure 3). The post hoc power analysis indicated that the test was sufficiently powered to detect this correlation (power = 0.887).

To compare electrolyte concentrations between patients who required surgery and those who did not, normality was reassessed based on surgical intervention. Sodium and chloride were not normally distributed, so the Mann–Whitney U test was applied. The test revealed no significant difference in sodium levels between non-surgical (mean rank = 20.15) and surgical patients (mean rank = 26.24) (U = 166.0, *p* = 0.125). However, a significant difference was observed in chloride levels, with non-surgical patients (mean rank = 14.26) having lower levels than surgical patients (mean rank = 21.79) (U = 68.5, *p* = 0.031). For potassium and calcium, which were normally distributed, independent-sample t-tests were conducted. Potassium levels were higher in non-surgical patients (mean = 4.01 mmol/L) than in surgical patients (mean = 3.75 mmol/L), but this difference did not reach statistical significance (t (42) = 1.825, *p* = 0.075, 95% CI [−0.028, 0.564], df = 42). Calcium levels were similar between non-surgical (mean = 1.035 mmol/L) and surgical patients (mean = 1.046 mmol/L), with no significant difference found (t (36) = −0.346, *p* = 0.732, 95% CI [−0.073, 0.052], df = 36).

**Figure 4:**
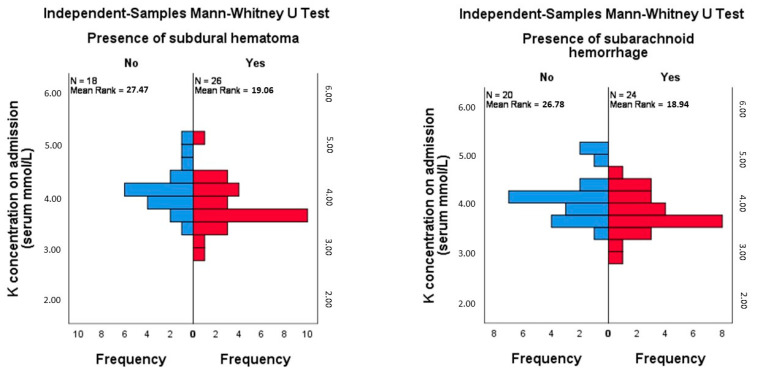
Mann–Whitney test comparison of mean rank of potassium levels in patients with and without subdural hematoma and with and without subarachnoid hemorrhage.

A significant difference in potassium levels was found between patients with and without subdural hematoma (Mann–Whitney U = 144.5, Z = −2.142, *p* = 0.032, r = −0.323), suggesting a moderate effect size. Patients without subdural hematoma had higher potassium levels (mean rank = 27.47) compared to those with subdural hematoma (mean rank = 19.06). A post-hoc power analysis for this comparison revealed a power of 0.57, indicating moderate power for detecting differences between these groups. Similarly, a significant difference in potassium levels was observed between patients with and without subarachnoid hemorrhage (Mann–Whitney U = 154.5, Z = −2.020, *p* = 0.043, r = −0.305). Patients without subarachnoid hemorrhage had higher potassium levels (mean rank = 26.78) compared to those with subarachnoid hemorrhage (mean rank = 18.94). The post hoc power for this comparison was calculated as 0.53 (Figure 4). No other significant differences were found between potassium levels and other imaging findings, nor were associations between the levels of other electrolytes and the imaging findings analyzed in the study (Table 2).

## 4. Discussion

Our study presents significant findings on the relationship between electrolyte disturbances and TBI within the first 24 h of admission. Specifically, we discovered an inverse association between chloride levels and GCS scores (ρ = −0.515; *p* = 0.002). This is a notable discovery, as chloride levels have not previously been a primary focus in TBI research and may offer new insights into patient management. However, our findings suggest that they may serve as a critical biomarker. Elevated chloride levels could indicate a more severe neurological status in TBI patients, thereby requiring close monitoring and possibly influencing therapeutic strategies. For instance, a study by Lee et al. found that hyperchloremia is linked to poor outcomes and increased mortality in major trauma patients, with hyperchloremia 48 h post-admission correlating with 30-day mortality [28]. In contrast, Jahanipour et al. reported no association between admission chloride levels and hospital mortality 24 h later [29].

Our findings underscore the early occurrence of electrolyte disturbances within the first 24 h post-trauma in TBI patients, providing critical insights for clinical management. Hyponatremia was present in 16% of patients, particularly in those with severe TBI, and could reflect early sodium dysregulation due to SIADH or CSWS [8,9,10,11,12]. Conversely, hypernatremia was observed in only 2% of cases, lower than typically reported in the literature, possibly due to the early timing of measurements before factors such as dehydration or hyperosmolar therapies became evident [5,6,7]. Hypokalemia affected 12% of patients, particularly those with moderate and severe TBI, likely resulting from catecholamine-induced potassium shifts and fluid losses, compounded by surgical interventions and resuscitation [14,16,17,18,19]. No cases of hyperkalemia were observed, possibly due to the early stage of injury assessment, before typical triggers like acidosis or transfusions could affect potassium levels [4,13,14,15,16]. Hyperchloremia, present in 22% of patients—especially those with severe TBI—suggests early disruptions in chloride regulation, which may contribute to metabolic acidosis and complicate neurological outcomes [25,26]. In contrast, hypochloremia was observed in 8% of patients, predominantly in those with milder injuries, indicating variability in chloride disturbances based on injury severity and early treatment. Finally, hypocalcemia was the most prevalent disturbance, affecting 64% of patients across all TBI severities, likely due to early transfusion-related calcium depletion and coagulopathy, both of which are associated with worse outcomes [22,23,24]. These findings emphasize the importance of early and vigilant electrolyte monitoring in TBI patients to guide timely therapeutic interventions and optimize patient outcomes.

Our research suggests that higher chloride levels correlate with lower GCS scores, potentially highlighting the importance of early chloride level monitoring. Several factors may contribute to elevated chloride levels in patients with severe TBI. Hyperchloremic metabolic acidosis can result from renal tubular acidosis, gastrointestinal losses, or iatrogenic causes [30]. Intravenous fluid therapy, particularly the use of normal saline which has a high chloride concentration, is often used in large volumes for initial resuscitation in severely injured patients [31]. Acute kidney injury, common in severely ill or injured patients, can impair chloride excretion, leading to accumulation [32]. Additionally, certain medications, such as carbonic anhydrase inhibitors, can cause hyperchloremia by reducing bicarbonate reabsorption in the kidneys [33].

In our study, patients who underwent surgery had significantly higher chloride levels compared to those who did not, suggesting that elevated chloride may not only correlate with lower GCS scores but also be associated with the severity of injury requiring surgical intervention. Our findings advocate for the routine monitoring of chloride levels in TBI patients from the time of admission. Early detection of hyperchloremia could enable timely interventions to address underlying causes and prevent potential complications. Careful consideration of fluid types and volumes could prove essential in the resuscitation of TBI patients. Alternatives to normal saline or strategies to mitigate hyperchloremia should be explored to optimize patient outcomes. Additional studies are necessary to explore the precise impact of chloride levels on TBI outcomes and to establish evidence-based guidelines for the management of electrolyte disturbances in these patients.

Our study found significant associations between potassium levels and the presence of subdural hematoma and subarachnoid hemorrhage, suggesting that potassium imbalances could be indicative of more severe brain injuries. However, no significant associations were found between imaging findings and sodium, ionized calcium, or chloride levels. This could indicate that while potassium levels may serve as a marker for certain types of brain injury, other electrolytes do not appear to have the same predictive value. Patients with subdural hematoma and subarachnoid hemorrhage had lower potassium levels compared to those without these conditions. This association has not been documented in the existing literature. However, lower potassium levels are frequently observed in severely traumatized patients. One explanation is that severely injured patients release higher quantities of adrenaline and cortisol, which increase renal potassium excretion and result in hypokalemia [14]. These patients often require aggressive fluid resuscitation to maintain blood pressure and cerebral perfusion. Administering large volumes of potassium-free intravenous fluids can dilute serum potassium levels. Additionally, diuretics, such as furosemide or mannitol, commonly used to reduce intracranial pressure, can lead to increased renal potassium loss [34,35].

Brain injuries can also cause renal dysfunction or inappropriate antidiuretic hormone release, resulting in cerebral salt wasting where the kidneys excrete excessive amounts of sodium and potassium. Furthermore, associated gastrointestinal disturbances like vomiting or diarrhea in brain injury patients can cause significant potassium loss [34,36]. Finally, potassium deficiency is often observed in cases of metabolic alkalosis, a condition common in severely injured patients. This deficiency can exacerbate the situation by promoting renal hydrogen ion secretion and increasing renal ammonium production and excretion [37].

Potassium and potassium channels have been shown to play an important role in subarachnoid hemorrhage and subdural hematoma. An animal study conducted by Chen et al. on rats demonstrated that activating large-conductance calcium-activated potassium channels in cerebral arteries could help alleviate spastic constriction [38]. Other studies have shown that subarachnoid hemorrhage-induced membrane potential depolarization, involving disrupted potassium homeostasis, leads to increased activity of voltage-dependent calcium channels, elevated smooth muscle cytosolic calcium levels, and subsequent parenchymal arteriolar constriction [39]. Experiments on small-conductance calcium-activated potassium channels have shown that blockers of KCa3.1 can reduce infarct volume in a rat subdural hematoma model. These findings suggest that potassium channels could become therapeutic targets for treating traumatic and potentially ischemic brain injury [40]. Additionally, there is significant evidence indicating that the mitochondrial ATP-dependent potassium channel plays a crucial role in the neuroprotective effects of cerebral preconditioning in a rat model of acute subdural hematoma [41]. These insights underscore the importance of potassium and maintaining its normal levels. They also highlight the intricate molecular mechanisms at play and the necessity of further investigations to understand why subdural hematoma and subarachnoid hemorrhage are associated with lower potassium levels, unlike other types of brain injuries. 

Our study revealed no significant association between sex and sodium, ionized calcium, or chloride levels in patients with TBI. However, a significant association was found between sex and potassium levels, with males exhibiting higher levels compared to females (*p* = 0.016, mean for males = 3.99 mmol/L, mean for females = 3.51 mmol/L). This finding may be influenced by several physiological factors. Firstly, differences in sex hormones, such as testosterone and estrogen, play a crucial role in potassium regulation, with testosterone potentially contributing to higher potassium levels in males. Additionally, males generally have greater muscle mass, which serves as a primary reservoir for potassium, thereby explaining the elevated levels observed. Furthermore, lipid peroxidation, which is more prominent in males following severe TBI, may also contribute to these differences by affecting cell membrane integrity and potassium homeostasis [42]. Our study’s limited female representation reflects the historical view of TBI as predominantly affecting males, due to their higher participation in high-risk activities. This male bias is also prevalent in preclinical research, where male animals are often the subjects of basic and translational studies. However, with an aging population and increasing female involvement in high-risk activities, the incidence of TBI is becoming more sex-independent. Therefore, the importance of recognizing and addressing sex differences in TBI responses and outcomes is paramount. Future research should focus on increasing female representation in studies and exploring sex-specific mechanisms in TBI to enhance the design of clinical trials and treatment strategies [43].

One of the strengths of this study is its focus on the first 24 h post-injury, providing a critical timeframe for understanding electrolyte changes in TBI patients. Another strength lies in the robust statistical power for key findings. The power analysis conducted for the significant correlation between chloride levels and GCS scores demonstrated that our study had adequate power (88.7%) to detect this effect, further supporting the robustness of this finding. Even though this focus on the early post-injury period provides valuable insights, it also introduces a limitation: the variability in the time between injury and admission, as patients were admitted at different times following the trauma. This variability, inherent to real-world clinical practice in a regional trauma center, could introduce some heterogeneity in the electrolyte measurements, particularly for patients who received initial stabilization at smaller hospitals before transfer. Although we limited our analysis to patients who arrived within 24 h of the trauma, this variation in admission times and prior care may have influenced the results. Nevertheless, this approach still provides meaningful insights into the early phase of electrolyte disturbances post-TBI. Additionally, the relatively small sample size and single-center design may limit the generalizability of the result. Power analysis for the significant associations in our study, such as those involving potassium levels and subdural hematoma or subarachnoid hemorrhage, indicated moderate power (0.53–0.57). This suggests that while we detected significant differences, the study may still be underpowered to detect smaller effects or subtle variations in electrolyte imbalances. Future research should aim to include larger, multi-center studies to validate our findings and provide a more comprehensive understanding of electrolyte imbalances in TBI patients. In addition, the observed sex distribution, with fewer female participants, mirrors real-world epidemiological data, where males show a higher incidence of TBI (388 per 100,000 for males versus 195 per 100,000 for females) [44,45]. This sex imbalance may affect the generalizability of our findings regarding electrolyte disturbances in female patients and underscores the importance of conducting future studies with more balanced sex representation. Longitudinal studies with longer follow-up periods would help determine the long-term effects of these imbalances and the efficacy of different treatment protocols.

## 5. Conclusions

Our study highlights the importance of monitoring electrolyte levels in TBI patients within the first 24 h post-injury. Elevated chloride levels were inversely related to GCS scores, suggesting that chloride could serve as a biomarker for severe neurological impairment. Additionally, lower potassium levels were associated with subdural hematoma and subarachnoid hemorrhage, indicating the potential severity of these injuries. Routine early monitoring of chloride and potassium levels can improve patient management and outcomes. Further research with larger, multi-center studies is necessary to validate these findings and develop evidence-based guidelines for managing electrolyte imbalances in TBI patients. Understanding the role of electrolytes in TBI will help enhance patient care and improve outcomes.

## Figures and Tables

**Table 1:** Distribution of electrolyte imbalances tested within 24 h of trauma.

Electrolyte Imbalance	Number of Patients (%)	TBI Severity	Surgical Intervention
Hypernatremia	1 (2%)	1 moderate TBI	0 surgery
Hyponatremia	8 (16%)	1 severe TBI, 2 moderate TBI, 5 mild TBI	1 surgery 7 no surgery
Hypokalemia	6 (12%)	1 severe TBI, 3 moderate TBI 2 mild TBI	2 surgery 4 no surgery
Hyperkalemia	0 (0%)	—	—
Hypochloremia	4 (8%)	1 moderate TBI 3 mild TBI	0 surgery
Hyperchloremia	11 (22%)	5 severe TBI 2 moderate TBI 3 mild TBI	8 surgery 3 no surgery
Hypocalcemia	32 (64%)	12 severe TBI, 6 moderate TBI 14 mild TBI	13 surgery 19 no surgery
Hypercalcemia	0 (0%)	—	—

**Table 2:** Association between electrolyte levels and imaging findings in patients with TBI (Mann–Whitney U Test).

		Electrolytes (*p* Values)			
		Sodium	Potassium	Ionized Calcium	Chloride
Imaging Findings	Basal skull fracture	0.070	0.128	0.946	0.606
	Other skull fractures	0.790	0.213	0.772	0.091
	Subdural hematoma	0.315	0.032	0.375	0.204
	Epidural hematoma	0.490	0.859	0.225	1.000
	Subarachnoid hemorrhage	0.140	0.043	0.849	0.870
	Intracerebral hematoma	0.717	0.322	0.827	0.250
	Diffuse axonal injuries	0.273	0.591	0.263	0.967

## Data Availability

The data presented in this study are available on request from the corresponding author. The data are not publicly available due to ethical restrictions.
